# Left nucleus accumbens volume is associated with poor sleep in hip osteoarthritis^☆^

**DOI:** 10.1016/j.ynpai.2025.100203

**Published:** 2025-11-13

**Authors:** Natalia Egorova-Brumley, Luiza Bonfim Pacheco, Gabby Knox, Leila Nategh, Fiona Dobson, Michelle Hall

**Affiliations:** a Melbourne School of Psychological Sciences, University of Melbourne, Victoria, Australia; b School of Psychological Sciences, Monash University, Melbourne, Australia; c Department of Physiotherapy, Melbourne School of Health Sciences, University of Melbourne, Victoria, Australia; d Sydney Musculoskeletal Health, The Kolling Institute, The University of Sydney, Sydney, New South Wales, Australia; e Musculoskeletal Research Hub, Charles Perkins Centre, The University of Sydney, Sydney, New South Wales, Australia; f Florey Institute of Neuroscience and Mental Health, Heidelberg, Victoria, Australia

**Keywords:** Nucleus accumbens, Sleep, Pain, Hip osteoarthritis, Chronic pain

## Abstract

**Objective:**

Reduction in the volume of the nucleus accumbens (NAc) has emerged as a promising signature of chronic pain transition, including in osteoarthritis (OA). Yet, less is known about the factors that could influence these changes in the mesolimbic system. Given that poor sleep is common in OA, and recent studies of sleep disturbance on pain perception in animals and healthy populations have specifically implicated the NAc, we hypothesised that the left NAc volume in hip OA would be associated with sleep quality and quantity. Furthermore, we explored how sex interacts with this relationship.

**Methods:**

Cross-sectional study in participants with hip OA (n = 34; aged 60+/-12 years, 67 % females) who reported moderate pain were recruited.

**Results:**

A one-sample *t*-test showed that the left NAc volumes were significantly lower than normative values (t = -2.7368, df = 33, p-value = 0.009). In a model (F(4, 29) = 6.642, p < 0.001, R2 = 0.4781) including the total intracranial volume (TIV), sex and age, the left NAc volume was significantly predicted by sleep quality (t = -3.416, p = 0.002) assessed with Pittsburgh Sleep Quality Index (PSQI). Sleep efficiency (t = 2.362, p = 0.025) but not hours spent in bed (p > 0.05) was also a significant predictor. With models exploring sleep*sex interactions, only sleep efficiency demonstrated an interaction, suggesting that the left NAc volume is lower in females with worse sleep efficiency (t = -2.086, p = 0.046, albeit not significant when corrected for multiple comparisons, pFDR = 0.138.)

**Conclusions:**

Our results suggest that the reduction of the NAc volumes as a candidate biomarker of pain might be influenced by sleep. This exploratory finding in a chronic hip OA population is consistent with the results previously only reported in animal and experimental pain/sleep studies in healthy participants.

**Significance:**

The left NAc as a candidate biomarker of chronic pain is sensitive to the effects of sleep quality, especially in females with OA.

## Highlights

• The left NAc as a candidate biomarker of chronic pain is sensitive to sleep quality.
• Low sleep efficiency is related to lower left NAc volume particularly in females.
• The left NAc volume is lower in hip osteoarthritis relative to normative values.

## Introduction

The nucleus accumbens (NA) is a subcortical brain structure within the ventral striatum that is traditionally associated with reward and motivation, although it is also known to play a key role in pain processing (Baliki et al., 2010, Becerra et al., 2001, Navratilova and Porreca, 2014). Specifically, the left NAc slow-wave activity, connectivity and size (structural volume) have emerged as a promising signature of chronic pain transition (Makary et al., 2020), although both left and right structures have been implicated in pain (Baliki et al., 2012, Chang et al., 2014, Martucci et al., 2018, Vachon-Presseau et al., 2016).

Sleep disturbance is a known comorbidity of chronic pain (Haack et al., 2020, Mathias et al., 2018, Runge et al., 2024, Whale and Gooberman-Hill, 2022). Sleep quality and quantity are known to affect pain perception, with acute sleep disruption changing the way the brain processes pain, especially in the limbic system (Boakye et al., 2016). In experimental sleep studies in healthy participants, the NAc has been implicated in the effect of sleep deprivation and fragmentation on pain perception (Krause et al., 2019, Seminowicz et al., 2019). The NAc has been previously linked to sleep, particularly in preclinical models. A common neuronal ensemble in the NAc has been shown to regulate pain and sleep behaviour in mice (Sun et al., 2023). NAc has been implicated in the pronociceptive effect of sleep loss in rats via increasing the activity of adenosine A2A receptors transmission and decreasing the activity of dopamine D2 receptors (Sardi et al., 2018a, Sardi et al., 2024). Decreased alertness due to sleep loss was related to increased pain sensitivity in mice (Alexandre et al., 2017), which could be reversed with caffeine and modafinil, both related to the dopamine release in the NAc (Ferraro et al., 1996, Solinas et al., 2002).

Yet, whether sleep is a factor that modifies the structure and function of the NAc in people with chronic musculoskeletal pain remains unknown. We therefore aimed to understand if the volume of the left NAc as a specific biomarker of chronic pain (Makary et al., 2020) is affected by sleep quality and quantity.

Here we focused on osteoarthritis (OA), a chronic pain condition that is characterised by the complex combination of pain sensitization, nociceptive, inflammatory and neuropathic pain and may be influenced by psychosocial comorbidities, socioeconomic circumstances, immune alterations, hormonal changes, sleep impairments, and (epi)genetic features (Fauchon et al., 2025). Despite high heterogeneity, neuroimaging studies of OA are converging on a set of brain regions consistently and causally associated with OA, including the NAc (Barroso et al., 2025, Fauchon et al., 2025, Hall et al., 2023). Sleep disruption is characteristic of OA. Up to 31 % of OA patients report disturbances initiating sleep, 81 % have difficulties maintaining nighttime sleep (Parmelee et al., 2015). Poor sleep is both a risk factor for developing OA (Park et al., 2019) and a predictor of worse OA pain over time (Pan et al., 2020).

Accumulating evidence from experimental studies suggests that poor sleep might affect pain in a sex-dependent manner, with a stronger negative effect in females compared to males (Rouhi et al., 2023). Previous work has mostly been conducted in healthy young participants (Besedovsky et al., 2022, Haack et al., 2023), demonstrating stronger effects of sleep disturbance on central inhibitory processing in females (Rouhi et al., 2024a). Women also demonstrated reductions in the basal state of activation of the μ-opioid system during pain, specifically in the NAc (Zubieta et al., 2002). Much less is known about sex effects in the association between sleep and pain in clinical populations. Since OA has a high prevalence of sleep disturbance (Parmelee et al., 2015, Pickering et al., 2016, Smith et al., 2009, Song et al., 2021), and disproportionately affects females (Segal et al., 2024), we further aimed to explore whether the link between sleep and NAc volume in OA is stronger in females.

## Methods

This cross-sectional study was conducted at the University of Melbourne with the approval of the Human Research Ethics Committee (Ethics ID: 2021–21141-15536). All participants provided written consent in person in accordance with the Declaration of Helsinki. All testing and brain imaging were conducted at the Melbourne Brain Centre, Austin Hospital campus between 2022 and 2023.

### Participants

We recruited participants from the community across metropolitan Melbourne, Australia, through online advertisements (e.g., social media) and our volunteer database. Screening was conducted with an electronic survey (Qualtrics) and eligibility was confirmed by telephone. The inclusion criteria were: fulfilment of hip OA clinical criteria (NICE, 2022) (age ≥ 45 years, activity related hip pain, and no morning stiffness or morning hip stiffness ≤ 30 min); hip pain on most days for the past month; reported history of hip pain for more than 3 months; a mean overall hip pain severity score of 4 or greater over the past 1 week on an 11-point numerical rating scale (NRS; with terminal descriptors of ‘no pain’ [0] and ‘worst pain possible’ [10]) (Hawker et al., 2011). Sex was not controlled during recruitment – male and female participants were invited to take part in the study, no selection of participants was made based on sex. Participants were excluded if they were waiting for a back/lower limb surgery in the next 12 months, had previous hip replacement in the affected hip, had undergone any hip surgery in the past 6 months, were taking corticosteroids or had done so in the past 3 months, had any hip injection in the past 3 months or planned injection in the next 9 months.

### Pain and sleep characteristics

Patients reported their pain using the NRS (Hawker et al., 2011) and the Western Ontario and McMaster Universities Osteoarthritis Index (WOMAC) scales (Bellamy et al., 1988). Subjective sleep measures included the Pittsburgh Sleep Quality Index (PSQI) global score (Buysse Charles F Reynolds Ill et al., n.d.), and specifically differentiated between sleep efficiency and hours in bed.

Structural brain scans were obtained with 3 T MRI. A high-resolution magnetization-prepared rapid acquisition with gradient echo (MPRAGE) volume was acquired, with 160 sagittal slices and 1 mm isotropic voxels, repetition time (TR) = 1900 ms, echo time (TE) = 2.55 ms, 9° flip angle, 100 % field of view in the phase direction and 256 × 256 acquisition matrix. FreeSurfer 7 (https://surfer.nmr.mgh.harvard.edu/) subcortical segmentation with standard preprocessing was used to extract bilateral NAc volumes. The focus was on the left NAc in line with the previous study (Makary et al., 2020), however the right NAc was also examined.

### Statistical analyses

Although the current study did not compare NAc volumes to a control experimental cohort, a known normative average value of 429.67 mm^3^ from a previous study (Birbilis et al., 2021) that measured nucleus accumbens volumes in over N = 106 MRI scans of healthy adults (aged between 18 and 80 years, average age: 38.6 years; 67 women) was used. Note that while the average age in the current population was higher, Birbilis and colleagues explicitly explored the effect of age on the raw and normalised NAc volumes, finding no significant age effects on either, suggesting that their normative values were not age-dependent overall, but declined with age more in men than women.

We conducted a one-sample *t*-test, to determine whether there was a significantly reduced volume of the left NAc in the OA cohort.

We then explored the association between NAc volumes and sleep by conducting multiple regressions with the NAc volume as a dependent variable and sleep, total intracranial volume (TIV), sex and age as predictors. Separate analyses were conducted for PSQI (sleep quality), sleep efficiency and hours in bed. Original and FDR-adjusted p-values are reported to account for multiple comparisons in each of the ROIs. G*Power sensitivity analysis suggested that with N = 34 at p < 0.05, we had 80 % power to detect only large effects (f2 > 0.4), using our model with 4 predictors. These primary analyses focused on the left NAc volume but were also repeated for the right NAc. Additional sensitivity analyses including pain severity scores in the model were conducted.

Then we conducted an exploratory analysis including the sleep*sex interaction effect in the models, to identify whether sleep in males and females differentially affected the association with the left NAc volume. All analyses were 2-tailed and used p < 0.05 to assess significance.

## Results

Thirty-four participants with hip OA (aged 60+/-12 years, 23 females (67 %)) were recruited Participants reported pain of 5 (range 4–10) on a numeric rating scale (0–10). See Table 1 for the sample characteristics.

**Table 1:** Baseline characteristics of the sample.

**Variables**	
Age (years), SD	60, 12
Sex (females), %	23, 67 %
Height (m), SD	1.7, 0.1
Bodyweight (kg), range	85, 53–112
BMI (kg/m^2^), range	29.8, 21–40
Years of education (years), SD	13, 3
Hip symptom duration (years), SD	4.5, 5.3
PainDETECT score, SD	8.5, 5
WOMAC score, SD	36, 15
Unilateral symptoms, %	21, 62 %
PSQI, SD	8, 4
Sleep Efficiency, SD	77, 16
Hours in Bed, SD	8.6, 1.5
Left NAc volume SD	430, 93
Right NAc volume, SD	514, 81

SD – standard deviation; BMI: body mass index.

First, we confirmed whether there was a significant reduction in the left nucleus accumbens as a potential correlate of chronic pain. A one-sample *t*-test showed that left NAc volumes were significantly (∼10 %) lower in this sample relative to a normative value (t = -2.7368, df = 33, p-value = 0.009).

**Fig. 1:**
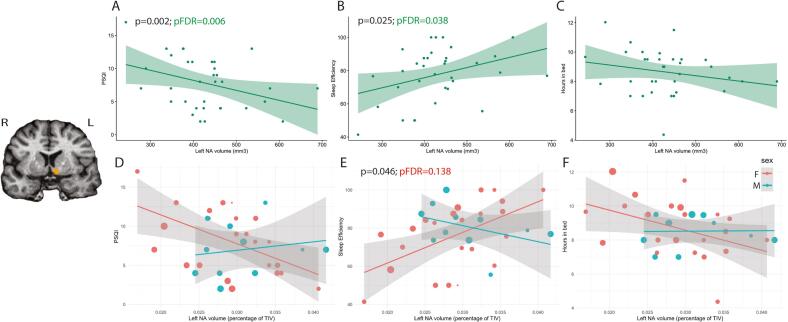
Main results. A. Correlation between the overall PSQI score and left NAc volume. B. Correlation between the Sleep Efficiency score and left NAc volume. C. Correlation between the Hours in bed and left NAc volume. D. Correlation between the overall PSQI and left NAc volume in males and females. E. Correlation between the Sleep Efficiency and left NAc volume in males and females. F. Correlation between the Hours in bed and left NAc volume in males and females. In plots D-F size of datapoints shows participant age (larger size = older age). Image shows the location of the left NAc volume in orange: L-left; R-right.

We then conducted a regression analysis to understand if sleep quality significantly predicted the volume of the left NAc in an adjusted model. This analysis resulted in a significant overall model, F(4, 29) = 6.642, p < 0.001, R2 = 0.4781, with PSQI (t = -3.416, p = 0.002), TIV (t = 2.413, p = 0.022) and age (t = -3.352, p = 0.002) emerging as significant predictors (Fig. 1). In addition, we conducted regression analyses separately for sleep efficiency and hours spent in bed. The analysis with sleep efficiency resulted in a significant model, F(4, 29) = 4.562, p = 0.005, R2 = 0.386, showing that sleep efficiency (t = 2.362, p = 0.025) and age (t = -2.372, p = 0.024) were significant predictors of the left NAc volume. However, analysis investigating the association with hours in bed resulted in a significant model, F(4, 29) = 3.178, p = 0.028, R2 = 0.305, where only the TIV was a significant predictor (t = 2.170, p = 0.038). With the FDR correction for multiple comparisons, the effects for PSQI (pFDR = 0.006) and sleep efficiency (pFDR = 0.038) remained significant.

The analyses conducted with the right NAc volumes did not reveal any significant effects or interactions involving any of the sleep variables. For PSQI, a significant overall model, F(4, 29) = 5.547, p = 0. 001924, R2 = 0. 433, showed no effect of PSQI (t = -0.678, p = 0.503) but a significant effect of sex (t = 2.936, p = 0.006) and age (t = -2.776, p = 0.009). For Hours in bed, a significant overall model, F(4, 29) = 5.45, p = 0.002, R2 = 0.429 showed no effect of Hours in bed (t = -0.486, p = 0.631) but significant sex (t = 2.741, p = 0.010) and age (t = -2.4, p = 0.023) effects. For Sleep Efficiency, a significant overall model, F(4, 29) = 5.593, p = 0.002, R2 = 0. 435 showed no significant effect of Sleep Efficiency (t = 0.751, p = 0.458), but a significant effect of sex (t = 2.928, p = 0.006) and age (t = -2.820, p = 0.008).

When pain severity (measured as 0–10 on the NRS) was added as an additional continuous predictor in the models that showed significant effects for the left NAc (PSQI and sleep efficiency), the results remained significant for both sleep efficiency (t = 2.108, pFDR = 0.044) and PSQI (t = -3.192, pFDR = 0.007). When pain severity was categorised as mild pain (0–4 on the NRS, with N = 15) and moderate-to-severe pain (5–10 on the NRS, with N = 19), there was no significant main effect of pain severity group, and the results remained significant for Sleep Efficiency (t = 2.207, pFDR = 0.036) and PSQI (t = -3.268, pFDR = 0.006). This suggests that observed sleep associations were not driven by the experience of pain.

Finally, we explored whether sex interacted with the effect of sleep on the left NAc volume, testing models with sleep*sex interaction included. A significant overall model, F(5, 28) = 4.943, p = 0.002, R2 = 0.469, including a significant sleep by sex interaction (t = -2.086, p = 0.046) was only observed for Sleep Efficiency, showing that the left NAc volumes were lower in females with worse Sleep Efficiency (Fig. 1). However, with the FDR correction for multiple comparisons, the effect for Sleep Efficiency was no longer significant pFDR = 0.138.

## Discussion

We investigated the association between the left NAc volume as a potential biomarker of chronic pain and sleep. We confirmed that, similar to a low back pain population (Makary et al., 2020), in our hip OA population the left NAc was lower than normative values. We acknowledge that that comparison to normative values rather than a matched control group is not optimal but note that this was not the primary aim of the study. In addition, we extend previous research to demonstrate that lower left NAc volume was associated with poorer sleep quality and lower sleep efficiency. In an exploratory analysis of the effect of biological sex on the sleep – NAc volume association, we observed that lower sleep efficiency had a stronger link with lower left NAc volumes in females, controlling for total brain volume. This set of findings confirms an important role of the NAc as a sleep-pain nexus, shedding new light on sleep as a potential factor influencing structural decline in this mesolimbic structure in chronic pain. Our results also tentatively suggest that females with hip OA might be more susceptible to the negative effects of poor sleep on the structure of the left NAc, but these results were not as robust (did not survive correction for multiple comparisons) and require replication in a larger study.

While the neurobiological changes associated with OA (Barroso et al., 2025, Fauchon et al., 2025, Hall et al., 2023) are widespread and complex, the current study emphasises the role of the emotional and motivational dopaminergic pain network in OA. Several lines of evidence have pointed to a critical role of the mesolimbic system, notably the nucleus accumbens (NA) and the dopamine system on the proalgesic effects of sleep loss on pain (Finan and Smith, 2013, Kourbanova et al., 2022, Sardi et al., 2018a). Dopamine is a putative mechanism for the link between chronic pain, depression and insomnia (Finan and Smith, 2013). There has been a call to shift focus in OA from primarily targeting the joint to incorporating both peripheral and central neuroplasticity-based interventions (Barroso et al., 2025). Our findings suggest that sleep might be further explored as an important modifable factor in OA pathophysiology, with sleep-focused treatments incorporated into clinical care for OA (Klyne et al., 2024). There is an increasing recognition of the effect of sleep on pain (Kourbanova et al., 2022b) with poor sleep exacerbating hip OA symptoms (Fu et al., 2019). Animal research has identified a common neuronal ensemble in NAc that regulates pain-like behaviour and sleep through divergent downstream circuit targets (Sun et al., 2023), and the current study attempts to bridge these presently disparate areas of focus in OA.

The NAc has been specifically linked to non-rapid-eye-movement (NREM) and slow wave sleep. For example, experimental activation of a select population of NAc neurons exacerbated pain-like (nociceptive) responses and reduced NREM sleep (Sun et al., 2023). A subset of NAc core neurons is known to specifically control slow-wave sleep, contributing to cognitive and emotional factors that influence sleep–wake behaviour (Oishi et al., 2017). It has been hypothesised that changes of infra-slow BOLD fluctuations in chronic pain might indicate impairment of slowly propagating waves (Ploner et al., 2017), with evidence supporting that resting state slow activity in awake state could be linked to resting state activity during slow wave sleep (Mitra et al., 2015). Therefore, previously observed changes in the infra-slow oscillations in the left NAc associated with pain (loss of power spectral density in the slow-5 frequency band (0.01 to 0.027 Hz)) (Makary et al., 2020) could indicate changes in slow wave sleep in our population. Future work needs to directly investigate the association between the NAc and slow wave sleep in OA. Our exploratory result demonstrating that the effect of sleep efficiency on the NAc volume varies by sex, with females showing a stronger association, is consistent with previous studies that identified that sleep-related dysfunction in pain inhibition (measured with conditioned pain modulation) particularly in women is specifically related to NREM and slow wave sleep (Rouhi et al., 2024b). Unlike the experimental studies in young healthy adults that focused on sleep duration (Rouhi et al., 2023), and in line with previous evidence of the role of sleep efficiency (Campbell et al., 2015), we show that it is sleep quality and sleep efficiency rather than time spent in bed that was associated with the NAc volume. Given the OA population is typically older, the impact of sleep restriction (e.g., due to work) is likely reduced. In contrast, difficulty initiating and maintaining sleep and sleep fragmentation, due to the experience of pain at night along, with the resulting changes in sleep architecture are more relevant and likely to impact sleep quality more than quantity (Reid et al., 2023).

## Limitations and future directions

Given the relatively small sample used here, we only focused on a specific hypothesis about the relationship between sleep and the left NA. Larger studies investigating the whole brain are needed to fully characterise the network of brain regions impacted by poor sleep in chronic pain. Equally, the analysis of sex effects performed here was exploratory only (not a priori powered). This research question should be studied further in bigger samples. Large neuroimaging datasets, such as ENIGMA Chronic Pain (Quidé et al., 2024) could be used to extend the research findings reported here.

The structural data in this study was obtained with the standard 3 T scanner and we extracted volumes of the whole NA, disregarding the distinction between NAc shell vs. core (Makary et al., 2020). While it is in principle possible to segment these at 3 T, future work using higher-resolution MRI is needed to elucidate the specific effect of sleep on NAc substructures.

The current study was observational and cross-sectional, allowing us to only demonstrate associations between sleep and NAc volume, not identify causal effects. The directionality of effects needs to be further established – whether poor sleep predates or follows from the changes in NAc cannot be determined from the current study.

We observed effects in the left NAc volume, consistent with the previous study reporting the left NAc volume decrease and the loss of slow frequency fluctuations (Makary et al., 2020) associated with pain. However, the evidence for strong lateralisation in pain is lacking, with many previous studies reporting bilateral involvement of the NAc in pain and reward processing (Baliki et al., 2012, Chang et al., 2014, Martucci et al., 2018, Vachon-Presseau et al., 2016). Future studies should investigate whether there are systematic differences between the left and right NAc with respect to sleep in a fully powered study.

Finally, we here reported the association of the NAc with sleep, which was measured using PSQI questionnaires. Objective sleep tracking in this population is needed to clarify whether perceived sleep quality matches objectively recorded sleep. Furthermore, while we hypothesise the link with slow wave sleep based on previous clinical and preclinical literature, the specific association between slow wave sleep and the left NAc in the current study cannot be established. Future studies with polysomnography (PSG) or electroencephalography (EEG)-based objective sleep tracking to establish sleep quality with respect to specific sleep stages, are needed to validate these hypotheses.

## Conclusions

Our results add the accumulating evidence that the left NAc is a potential biomarker of chronic pain and provide preliminary data to show that the volume is associated with poor sleep, with females showing a stronger link. These exploratory findings need to be confirmed, especially using study designs that can demonstrate causal links between sleep, pain and NAc volume.

## CRediT authorship contribution statement

**Natalia Egorova-Brumley:** Writing – review & editing, Writing – original draft, Visualization, Supervision, Resources, Project administration, Funding acquisition, Formal analysis, Conceptualization. **Luiza Bonfim Pacheco:** Writing – review & editing, Project administration, Data curation. **Gabby Knox:** Writing – review & editing, Project administration, Data curation. **Leila Nategh:** Writing – review & editing, Formal analysis. **Fiona Dobson:** Writing – review & editing, Resources, Funding acquisition, Conceptualization. **Michelle Hall:** Writing – review & editing, Resources, Funding acquisition, Conceptualization.

## Declaration of competing interest

The authors declare the following financial interests/personal relationships which may be considered as potential competing interests: Natalia Egorova-Brumley reports financial support was provided by Australian Research Council. If there are other authors, they declare that they have no known competing financial interests or personal relationships that could have appeared to influence the work reported in this paper.

## Data Availability

Data will be made available on request.
